# Supplementary material for: Allosteric modulation of ghrelin receptor signaling by lipids
Source: Nat Commun. 2021 Jun 24;12:3938. doi: 10.1038/s41467-021-23756-y (PMC8225672; doi:10.1038/s41467-021-23756-y)
Supplement: Supplementary file 1 — Supplementary Information [file 41467_2021_23756_MOESM1_ESM.pdf]

Supplementary Information

Allosteric modulation of ghrelin receptor signaling by lipids

Marjorie Damian, Maxime Louet, Antoniel Augusto Severo Gomes, Céline M'Kadmi, Séverine Denoyelle, Sonia Cantel, Sophie Mary, Paulo M. Bisch, Jean-Alain Fehrentz, Laurent J. Catoire, Nicolas Floquet, Jean-Louis Banères

	Ki (nM)		
	12 nm nanodiscs	17 nm nanodiscs	HEK293T
ghrelin	63.5 ± 8.2	72.1 ± 9.3	1.9 ± 0.3
JMV3011	4.5 ± 0.9	2.5 ± 0.7	1.0 ± 0.7
LEAP2(1-14)	6.7 ± 1.1	4.6 ± 0.5	3.6 ± 0.6

Supplementary Table 1. Insertion into large nanodiscs does not affect the ligand binding properties of GHSR. The 12 nm nanodiscs were obtained using the MSP1E3D1 scaffolding protein, as described[1]. The 17 nm nanodiscs were obtained with the cNW30 scaffolding protein. Ghrelin is the natural full agonist, JMV3011 a neutral antagonist[2] and LEAP2(1-14) the active N-terminal region of the natural inverse agonist LEAP2[3]. The values in HEK293T cells are from M'Kadmi et al.[2] (ghrelin, JMV3011) and M'Kadmi et al.[3] (LEAP2(1-14)). To be noted, the Ki values obtained for the full agonist ghrelin are higher in both kinds of nanodiscs than in HEK cells, consistent with the fact that the isolated receptor uncoupled from its cognate G protein is in a low affinity state for agonists. We previously demonstrated that high-affinity agonist binding can be restored by adding isolated Gq to the purified receptor[4].

	Bodipy-FL PIP2			Bodipy-FL PA		
% labeled lipid	emission intensity (520 nm)	concentration (μM)	lipid/receptor molar ratio	emission intensity (520 nm)	concentration (μM)	lipid/receptor molar ratio
0.5	118.63	0.6	1.2	109.99	0.6	1.2
1	220.24	1.1	2.2	204.42	1.2	2.4
1.5	331.16	1.7	3.4	299.16	1.8	3.6
2	442.10	2.3	4.6	393.69	2.4	4.8
2.5	552.85	3	6	495.96	3.2	6.4
3	654.68	3.3	6.6	582.62	3.7	7.4

Supplementary Table 2. Fluorescent lipid content of the nanodiscs. The fluorescent lipid concentrations were calculated from the emission intensity at 520 nm (λ_{exc} = 505 nm) of the nanodiscs used in the FRET assay using the calibration curves in Supplementary Figure 2b,c. The % of labeled lipid is the molar ratio of Bodipy-FL PIP2/PA-to-total lipid in the mixture before nanodisc assembly expressed in %. The lipid-to-receptor ratio was calculated from the GHSR concentration in the nanodiscs used in the FRET assay, *i.e.* 0.5 μM.

	GP
DMoPC	-0.017 ± 0.009
DOPC	-0.018 ± 0.007

Supplementary Table 3. DMoPC and DOPC nanodiscs have similar membrane fluidity. Laurdan general polarization (GP) for DMoPC and DOPC nanodiscs. Laurdan was incorporated at a 1:200 molar ratio into empty nanodiscs composed of either DMoPC or DOPC. The emission spectrum of the probe was acquired at 15°C between 400 and 600 nm with an excitation set at 358 nm (bandwidth 5 nm). GP was calculated from the emission intensities at 440 and 490 nm ($GP=(I_{440}-I_{490})/(I_{440}+I_{440})$)[5].

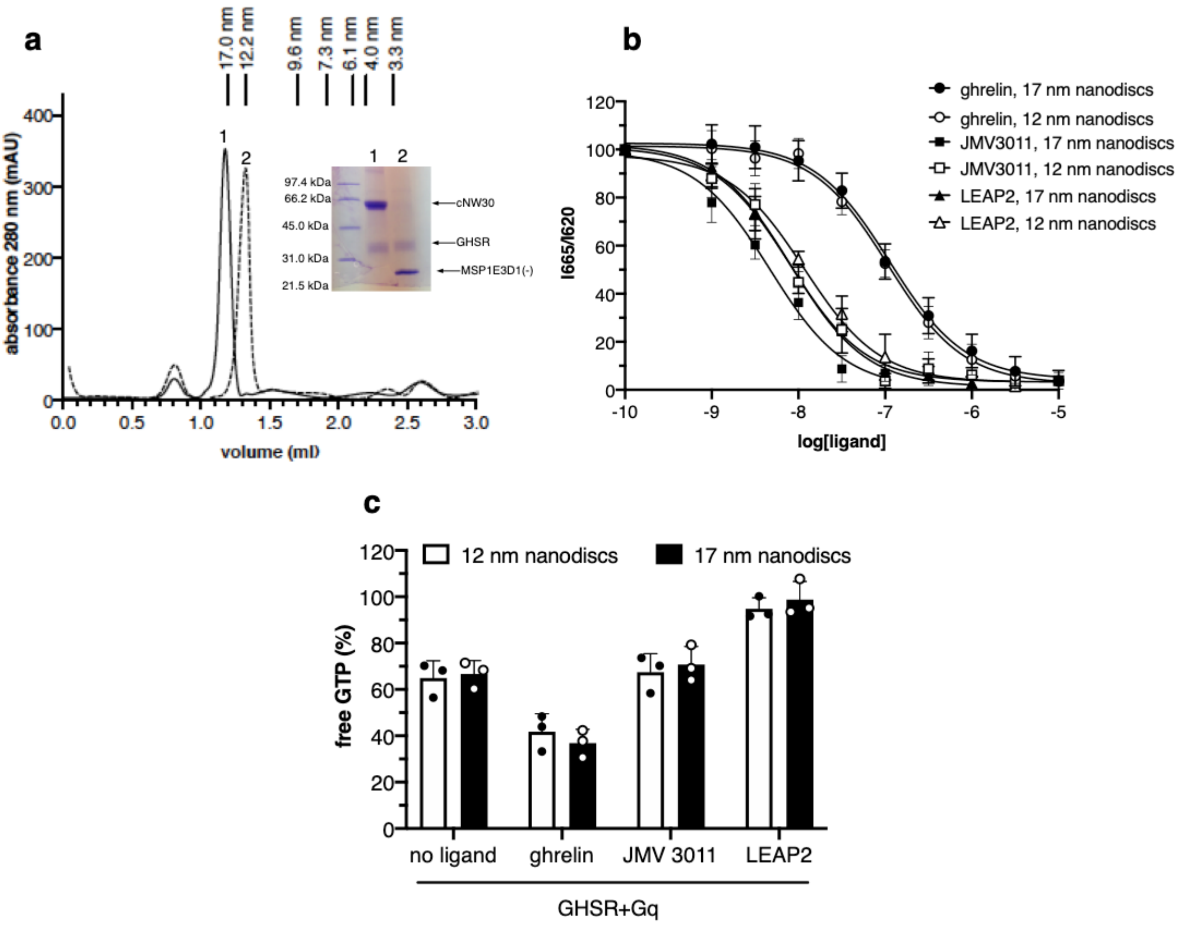

Supplementary Figure 1. Insertion into large nanodiscs does not affect GHSR pharmacological properties. (**a**) Size-exclusion profile obtained for GHSR-containing nanodiscs formed with cNW30 (plain line) or MSP1E3D1(-) (dotted line). The nanodiscs were run on a S200 increase column (5x150) using a 25 mM HEPES, 150 mM NaCl, 0.5 mM EDTA, pH 7.5 buffer as the eluent and a 0.2 mL/min flow rate. Inset: SDS-PAGE profile of the GHSR-containing nanodiscs. The samples were run on a 12% polyacrylamide-0.1% SDS gel with Coomassie blue staining. (**b**) Competition between a fluorescent ghrelin peptide[6] and the full agonist ghrelin, the neutral antagonist JMV3011 or the inverse agonist LEAP2(1-14) for binding GHSR inserted into cNW30 (17 nm) or MSP1E3D1(-) (12 nm) nanodiscs. (**c**) GTP turnover for Gq in the absence of ligand or in the presence of 10 µM ghrelin, JMV3011 or LEAP2(1-14) catalyzed by GHSR in cNW30 and MSP1E3D1(-) nanodiscs. The signal was normalized to that obtained for isolated G protein. Data in (**b**) and (**c**) is mean ± SD of three experiments, and source data provided as a Source Data file.

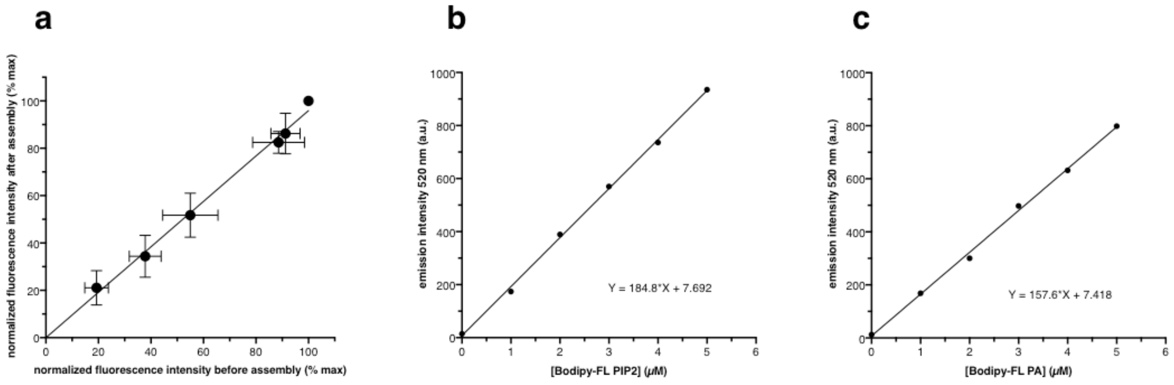

Supplementary Figure 2. PIP2 content after nanodisc assembly. (**a**) Normalized Bodipy-FL fluorescence intensity (λ_{exc} = 505 nm; λ_{em} = 520 nm) in the receptor:lipid:cNW30 mixture before and after nanodisc assembly from lipid mixtures containing increasing contents in Bodipy-FL PIP2, ranging from 0 to 3% (PIP2-to-total lipid molar ratio expressed in % ; see Supplementary Table 2). Data is mean ± SD of three experiments. Variations in fluorescence intensity as a function of Bodipy-FL PIP2 amount increased linearly before and after detergent removal and nanodisc assembly. This suggests that the amount of PIP2 in the discs after nanodisc assembly reflects that in the initial lipid mixture. (**b,c**) Calibration curves for Bodipy-FL PIP2 (**b**) and Bodipy-FL PA (**c**). These curves were obtained by measuring the emission intensity at 520 nm (λ_{exc} = 505 nm) of fluorescent lipid solutions of known concentration in a 25 mM HEPES, 150 mM NaCl, 0.5 mM EDTA, 100 mM β-DDM buffer. The equation of the linear regression used to calculate the fluorescent lipid concentration in the nanodiscs (Supplementary Table 2) is given in each plot. Source data are provided as a Source Data file.

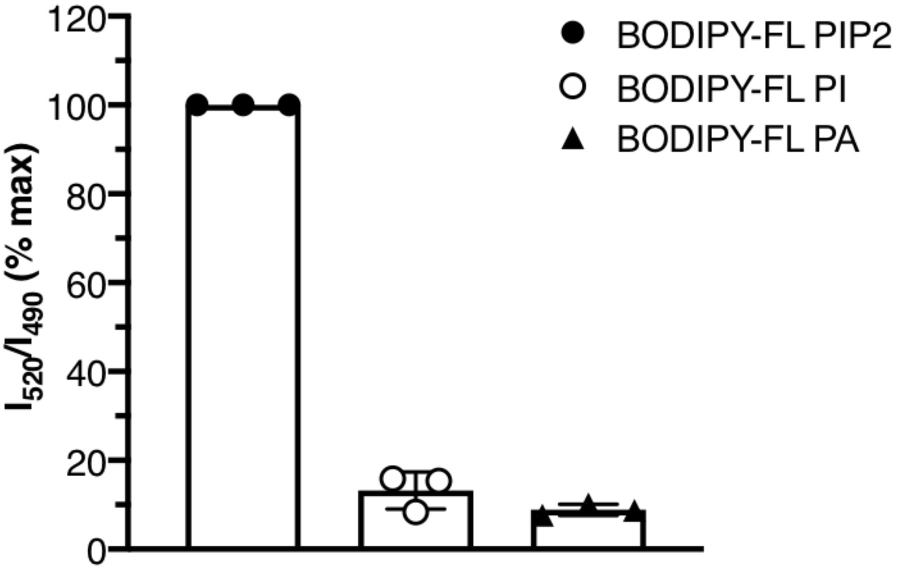

Supplementary Figure 3. The difference in FRET signal is not due to the difference in the structure of the acyl chain between Bodipy-FL PIP2 and PA. FRET signals in nanodiscs containing 2.5% Bodipy-FL PIP2, 2.5% Bodipy-FL PI or 2.5% Bodipy-FL PA and GHSR labeled with Lumi-4 Tb on C255$^{6.27}$. The signal was normalized to that obtained with Bodipy-FL PIP2 (first lane). Data are mean ± SD of three experiments. Source data are provided as a Source Data file.

a

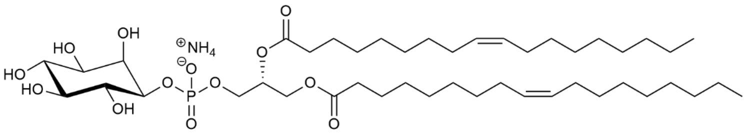

PI

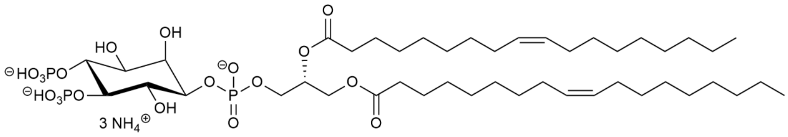

PI(4,5)P2

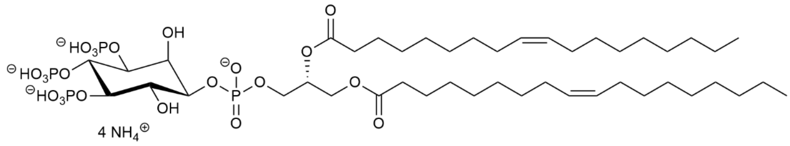

PI(3,4,5)P3

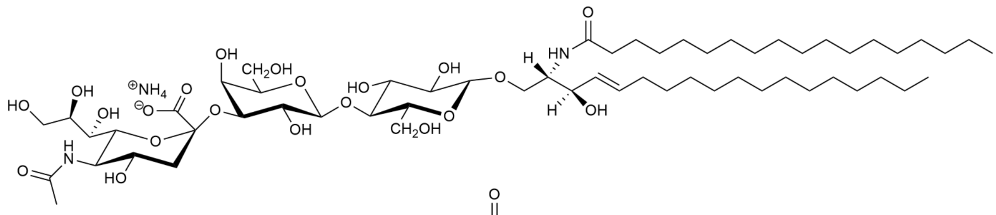

GM3

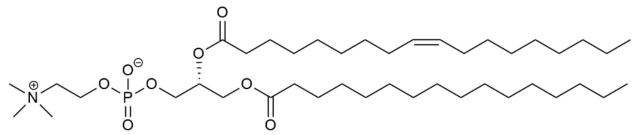

POPC

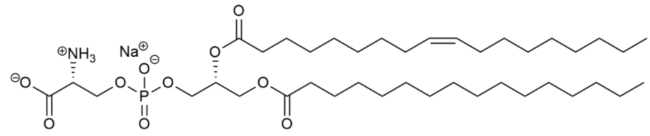

POPS

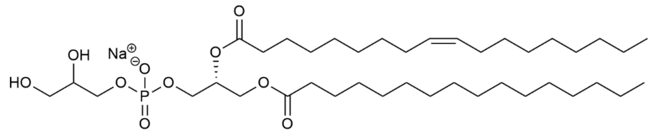

POPG

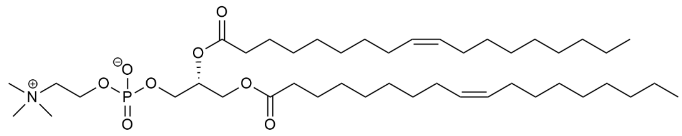

DOPC

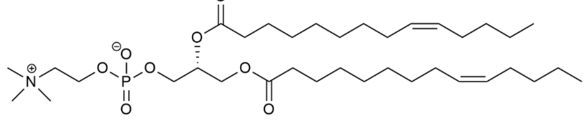

DMoPC

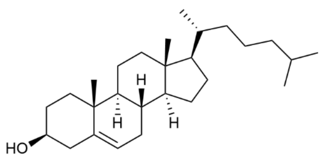

Cholesterol

b

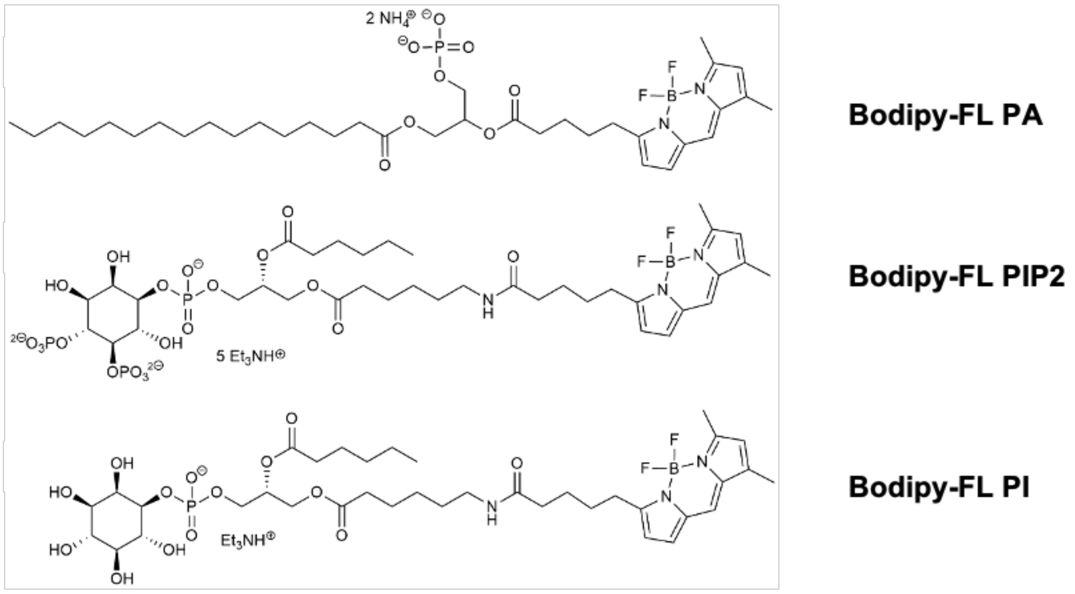

Supplementary Figure 4. Structure of the lipids (a) and their fluorescent derivatives (b) used throughout this work.

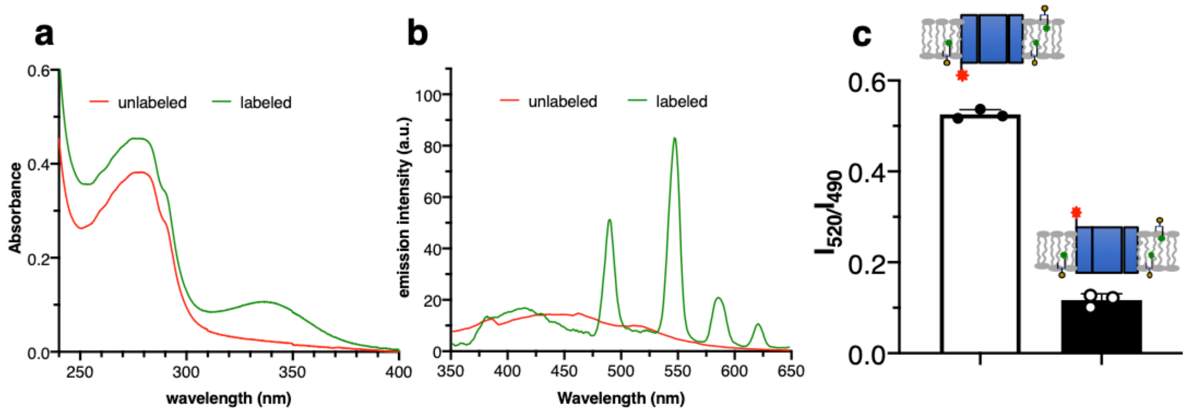

Supplementary Figure 5. PIP2 binding sites are likely located in the cytoplasmic region of the receptor. (**a**) UV absorption and (**b**) fluorescence emission spectra of GHSR bearing the unique reactive cysteine at position $304^{7.34}$ before (red line) and after (green line) labeling with Lumi-4 Tb maleimide. The fluorescence emission spectra were recorded with an excitation wavelength set at 337 nm, *i.e.*, at the maximum emission wavelength of the caged Tb. (**c**) FRET signal between GHSR and Bodipy-FL PIP2 (2.5 % Bodipy-FL PIP2-to-total lipid molar ratio) after excitation of Lumi-4 Tb at 337 nm. The Tb- donor was attached either to the cytoplasmic tip of TM6 ($C255^{6.27}$) or to the extracellular tip of TM7 ($C304^{7.34}$). Data in (**c**) is mean ± SD of three experiments and is provided as a Source Data file.

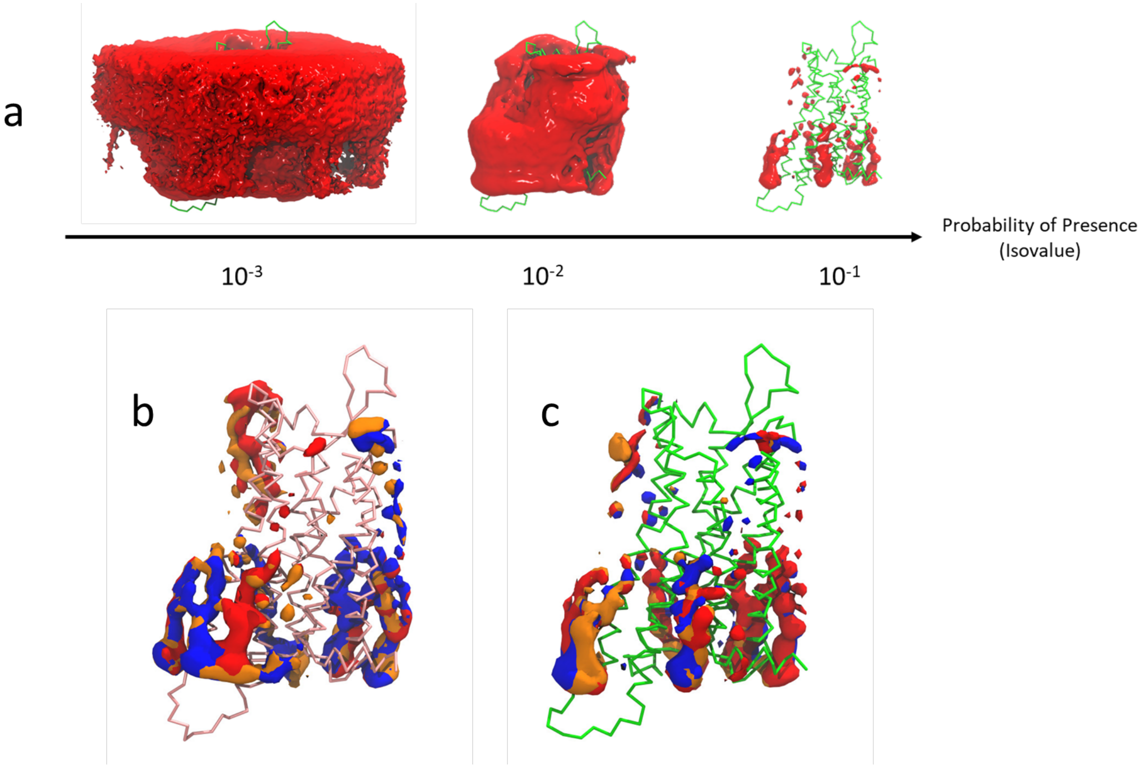

Supplementary Figure 6. Distribution of the PIP2 molecules around the GHSR as predicted by Coarse-Grained MD simulations. (a) Representation of the typical distribution of PIP2 observed along one of the CGMD simulations at different isovalues (red surface). This representation shows the preference for this lipid to bind to the intracellular side of the receptor. A finite number of 6 bound lipids is statistically observed for both the inactive (**b**, red ribbon) and the active (**c**, green ribbon) forms of the receptor, with a perfect reproducibility among the three replicas performed (surfaces reported in blue, orange and red, respectively, at an isovalue of 10^{-1}). All densities of PIP2 were computed from all the frames of the different simulations using the volmap tool implemented in VMD[7].

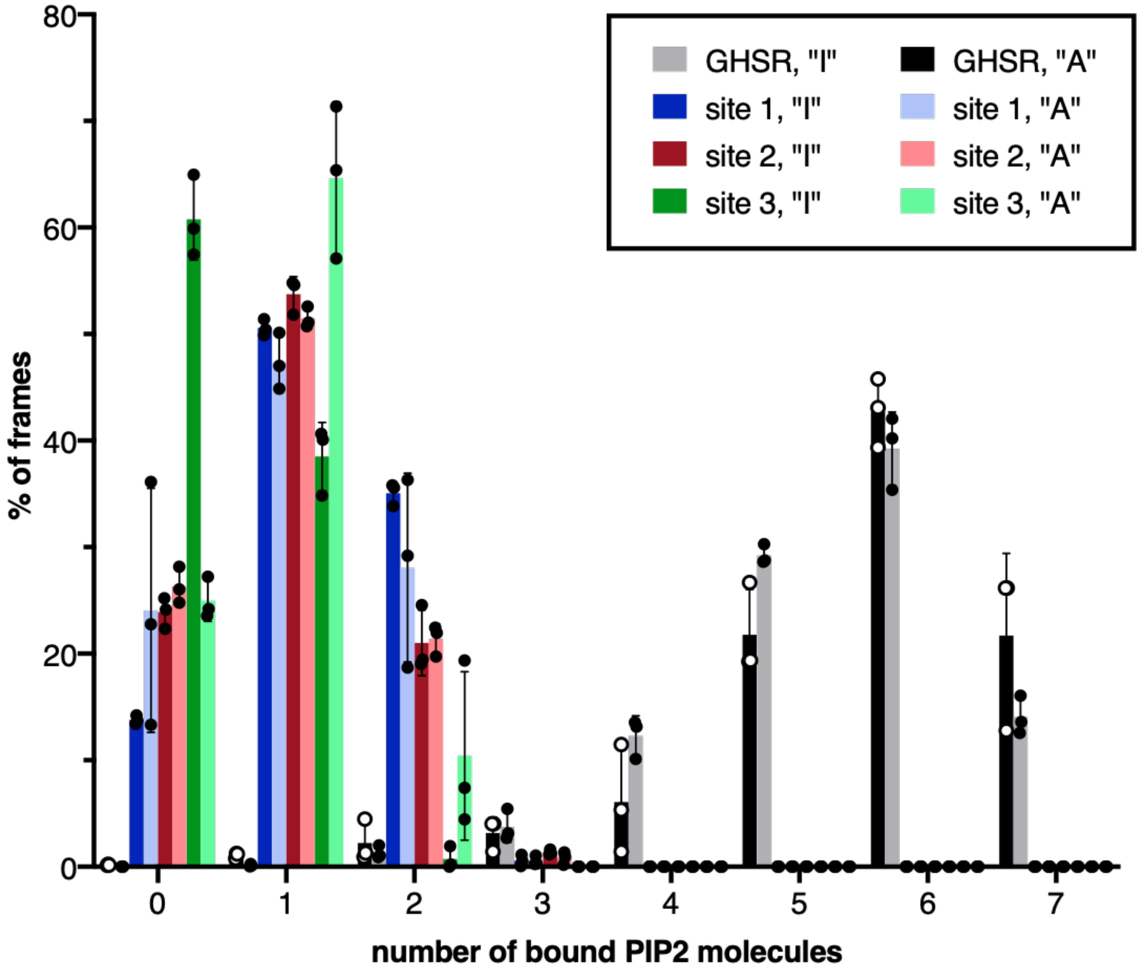

Supplementary Figure 7. Maximal number of PIP2 bound to GHSR in the CGMD simulations. Distribution of the number of PIP2 molecules bound to the intracellular side of GHSR inactive (“I”) and active (“A”) forms or to each of the three sites we identified in these simulations, *i.e.,* site 1 (blue), site 2 (red), and site 3 (green). Data are presented as mean ± SD computed among the three independent CG simulations. Source data are provided as a Source Data file.

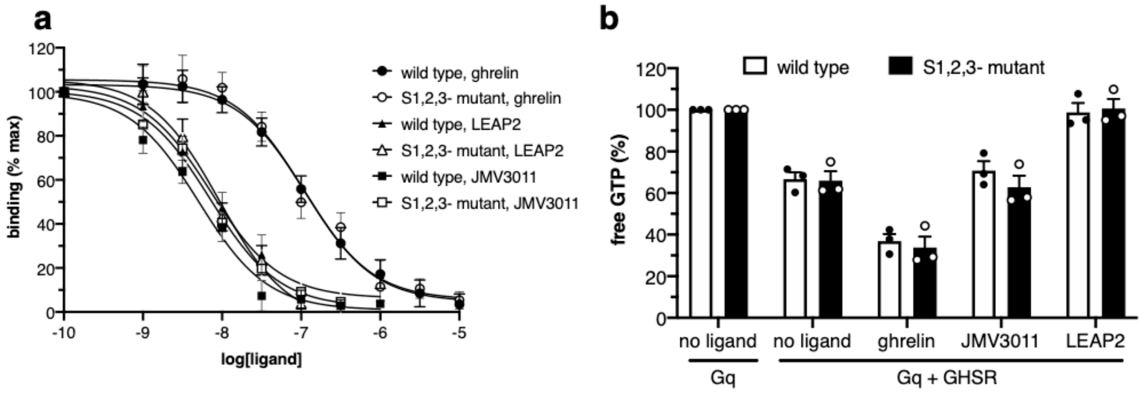

Supplementary Figure 8. The S1,2,3- mutation does not affect the pharmacological profile of GHSR in the absence of PIP2. (a) Competition between the fluorescent ghrelin peptide and ghrelin, JMV3011 or LEAP2(1-14) for binding GHSR or its S1,2,3- mutant in POPC nanodiscs. (**b**) GTP turnover for Gq catalyzed by GHSR or its mutant in POPC nanodiscs in the absence of ligand, in the presence of 10 µM ghrelin, of 10 µM JMV3011 or of 10 µM LEAP2(1-14). The signal was normalized to that obtained for the G protein alone. In all cases, data is mean ± SD of three experiments. Source data are provided as a Source Data file.

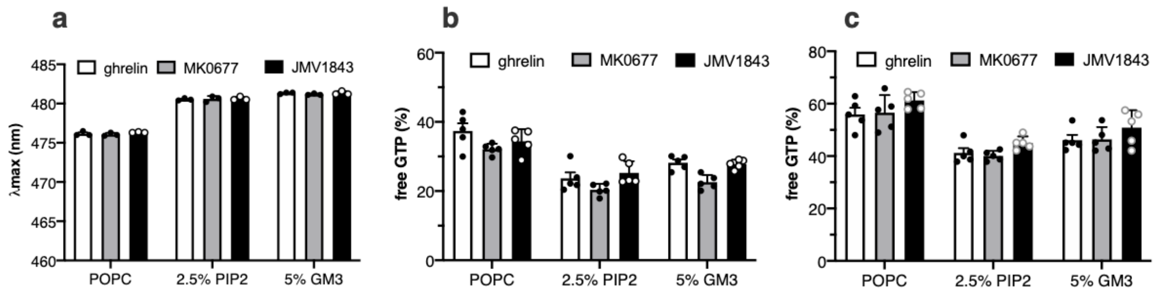

Supplementary Figure 9. The effects of PIP2 are not due to an interaction of the acyl chain of ghrelin with the bilayer. (**a**) λ_{max} of MB emission with GHSR in POPC, POPC:PIP2, POPC:GM3 nanodiscs in the presence of 10 µM of ghrelin, MK0677 or JMV1843. GTP turnover for Gq (**b**) and Gi2 (**c**) catalyzed by GHSR in POPC, POPC:PIP2, POPC:GM3 nanodiscs in the presence of 10 µM of ghrelin, MK0677 or JMV1843. The signal was normalized to that obtained for the G protein alone. Data in is mean ± SD of three (**a**) and five (**b**,**c**) experiments. Source data are provided as a Source Data file.

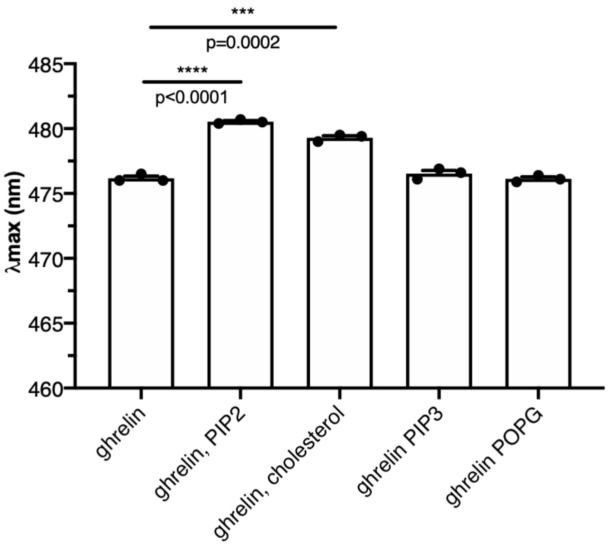

Supplementary Figure 10. PIP3 and POPG do not affect GHSR conformation, cholesterol likely does. λ_{max} of MB emission of GHSR in POPC, POPC:PIP2, POPC:PIP3, POPC:POPG or POPC:cholesterol nanodiscs in the presence of 10 µM ghrelin. In all cases, the POPC-to-additional lipid molar ratio was 5%. Data is mean ± SD of three experiments. Statistical values were obtained by means of unpaired Student's t test (***$0.0001<p<0.001$, ****$p<0.0001$). Source data are provided as a Source Data file.

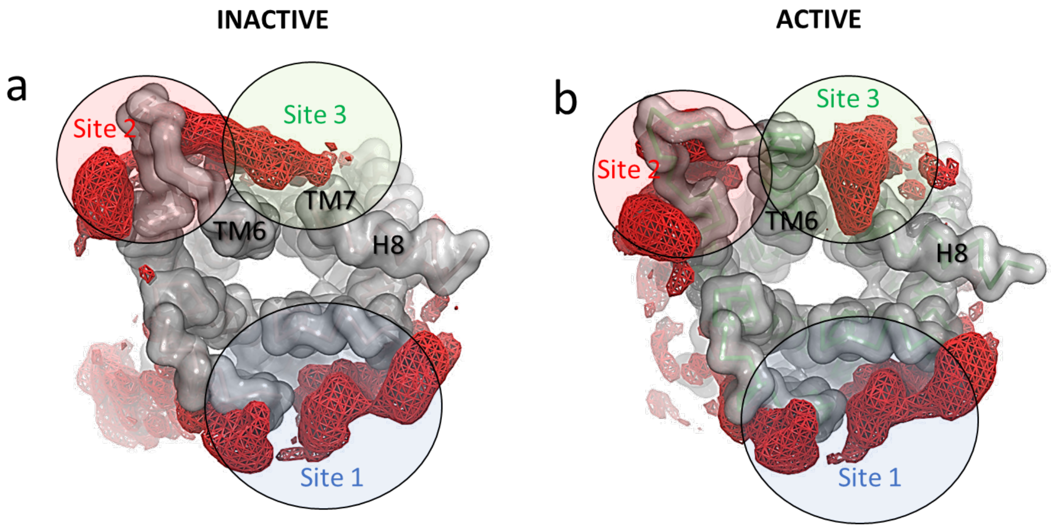

Supplementary Figure 11. Distribution of PIP2 around the inactive (a) and active (b) conformations of GHSR in the CGMD simulations. The three most occupied sites are reported with the same colors as in Supplementary Figure 6. The distribution of PIP2 is almost unchanged in sites 1 and 2 whereas a clear difference is observed in site 3 in which the PIP2 is intercalating between TM6 and TM7/H8. An isovalue of 10^{-1} was used and the reported densities are here the results of the three concatenated replicas in each case.

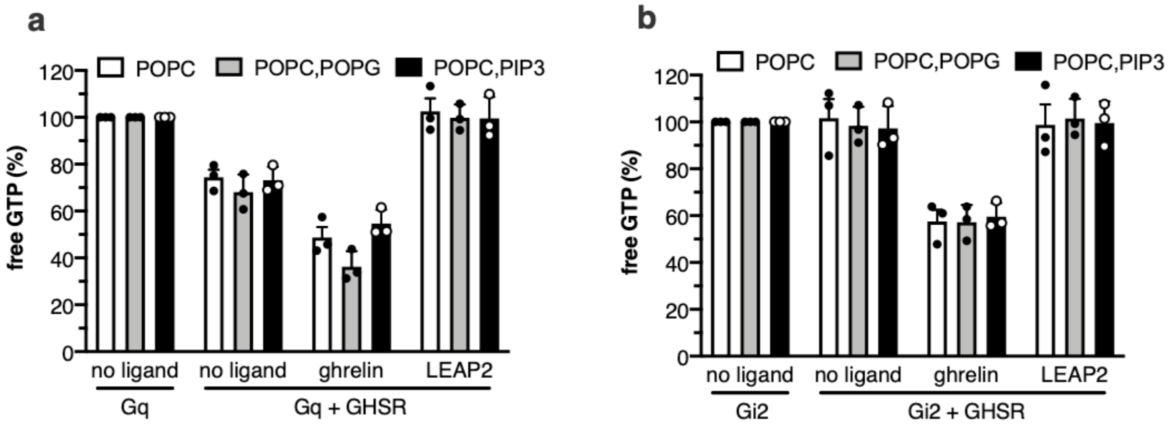

Supplementary Figure 12. POPG and PIP3 have no major effect on GTP turnover. GTP turnover for Gq (**a**) and Gi2 (**b**) catalyzed by GHSR inserted into cNW30 nanodiscs assembled with either POPC, POPC:POPG or POPG:PIP3 in the absence of ligand, in the presence of 10 µM ghrelin or in the presence of 10 µM LEAP2(1-14). In all cases, the POPC-to-additional lipid molar ratio was 5% to be comparable with PIP2. The signal was normalized to that obtained for the G protein alone. Data is mean ± SD of three experiments. Source data are provided as a Source Data file.

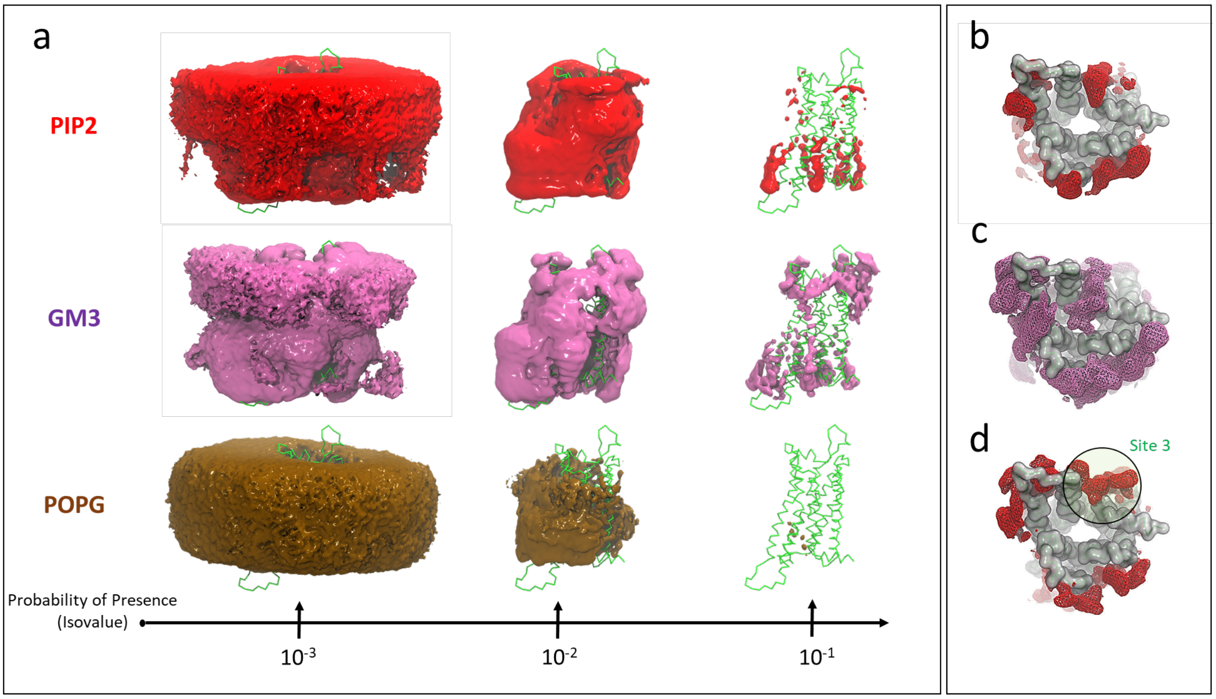

Supplementary Figure 13. Distribution of PIP2, GM3 or POPG around the GHSR active conformation in the CGMD simulations. (a) As compared to PIP2, GM3 binds indifferently to both sides of the receptor whereas no specific binding of POPG was observed at the same isovalue threshold. **(b,c)** On the intra-cellular side, GM3 binds to the same sites already identified for PIP2. This is confirmed by a redistribution of PIP2 once mixed to GM3 **(d)** mainly occurring in the site 3.

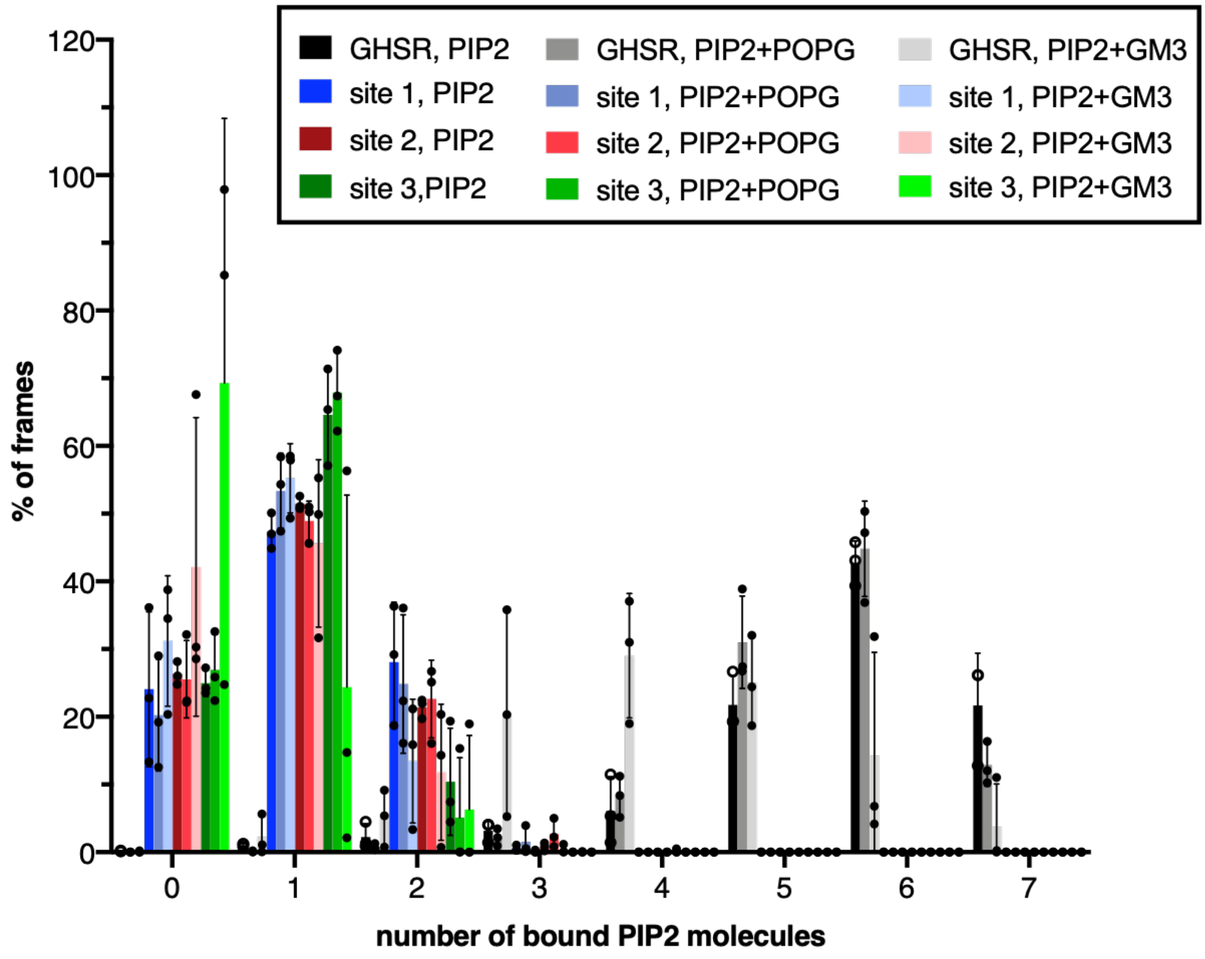

Supplementary Figure 14. Maximal number of PIP2 bound to GHSR active conformation in the CGMD simulations in absence or in the presence of GM3/POPG. No significant difference is observed with POPG (brown) whereas a shift to the left of the distribution of PIP2 is observed after addition of GM3 (purple). The site-by-site analysis (*i.e.,* site 1 (blue), site 2 (red), and site 3 (green)) confirms a main difference in site 3 after addition of GM3. Data are presented as mean ± SD of the three independent CG simulations performed for each condition. The large error bars observed in site 3 in the presence of GM3 are due to the fact that competition was observed in only 2 of the three performed simulations. Source data are provided as a Source Data file.

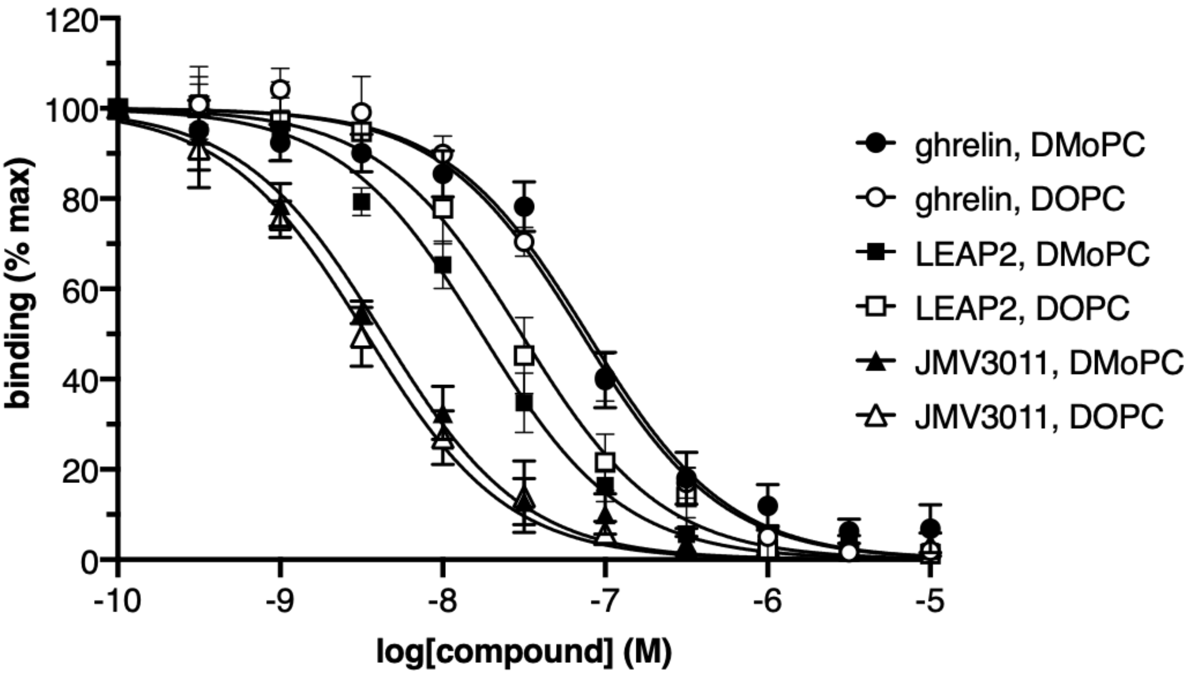

Supplementary Figure 15. Changing the membrane thickness slightly affects agonist and inverse agonist binding to GHSR. Competition between fluorescent ghrelin and ghrelin, JMV3011 or LEAP2(1-14) for binding GHSR assembled into DMoPC or DOPC nanodiscs. These binding assays were carried out in the absence of G proteins. Data is mean ± SD of three experiments. Source data are provided as a Source Data file.

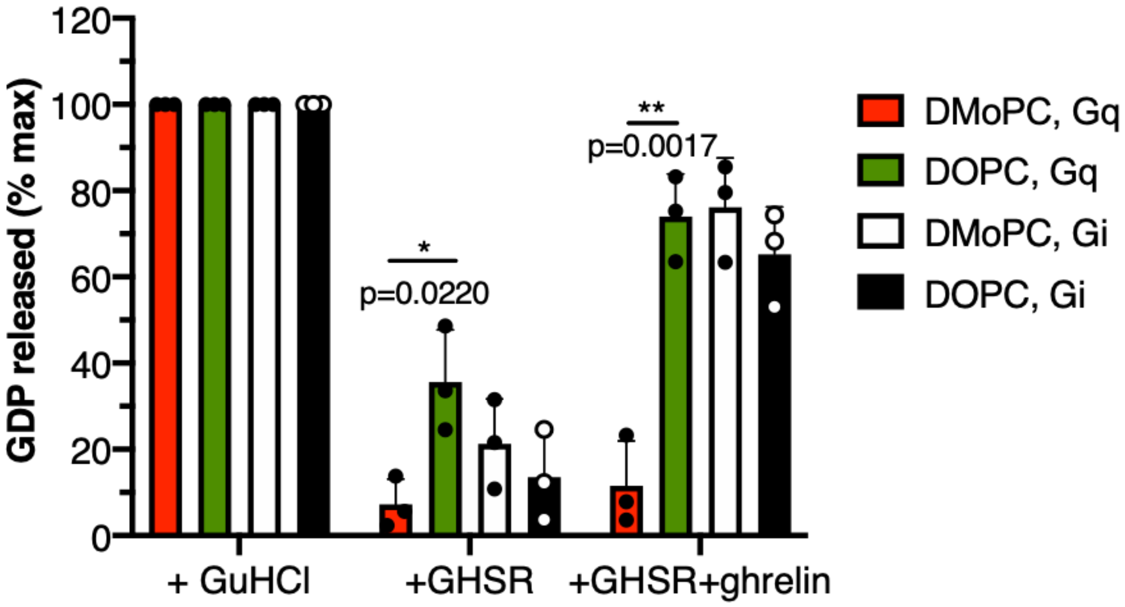

Supplementary Figure 16. Gq preassembled to GHSR inserted into DMoPC nanodiscs is likely in a GDP-loaded state. GDP release assay for Gq and Gi with GHSR assembled in either DMoPC or DOPC nanodiscs in the absence of ligand or in the presence of 10 µM ghrelin. Data was normalized to the amount of GDP released by G protein after chemical unfolding with guanidinium hydrochloride (+ GuHCl) and is the mean ± SD of three experiments. Statistical values were obtained by means of unpaired Student's t test (*0.01<p<0.05, **0.001<p<0.01). Source data are provided as a Source Data file.

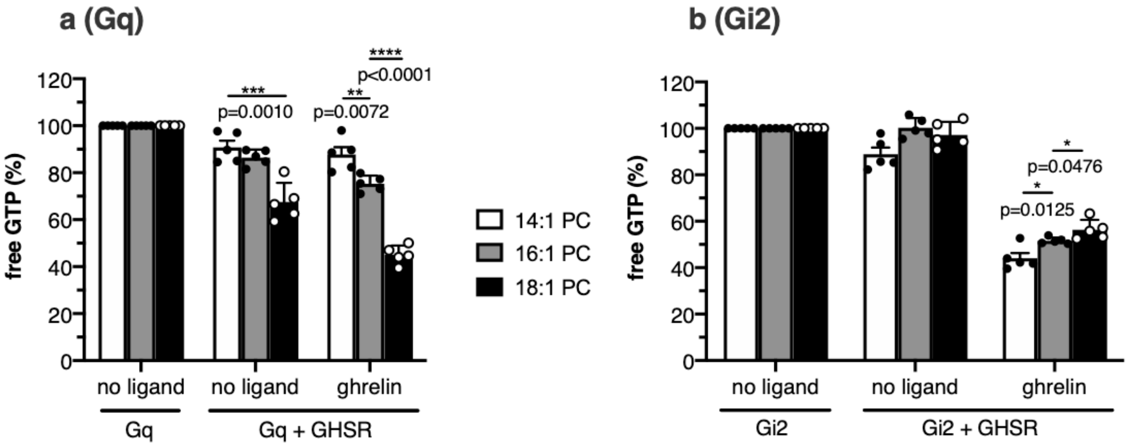

Supplementary Figure 17. Impact of membrane thickness on G protein activation. GTP turnover for Gq (**a**) and Gi2 (**b**) catalyzed by GHSR in 14:1 (Δ9-Cis) PC, 16:1 (Δ9-Cis) PC or 18:1 (Δ9-Cis) PC nanodiscs in the absence of ligand or in the presence of 10 µM ghrelin. Luminescent signal was normalized to the signal obtained for the G protein in the absence of receptor and ligand. Data is mean ± SD of five experiments. Statistical values were obtained by means of unpaired Student's t test (*0.01<p<0.05, **0.001<p<0.01, ***0.0001<p<0.001, ****p<0.0001). Source data are provided as a Source Data file.

Supplementary references

1. Damian M, *et al.* High constitutive activity is an intrinsic feature of ghrelin receptor protein: a study with a functional monomeric GHS-R1a receptor reconstituted in lipid discs. *J Biol Chem* **287**, 3630-3641 (2012).

2. M'Kadmi C, *et al.* Agonism, Antagonism, and Inverse Agonism Bias at the Ghrelin Receptor Signaling. *J Biol Chem* **290**, 27021-27039 (2015).

3. M'Kadmi C, *et al.* N-Terminal Liver-Expressed Antimicrobial Peptide 2 (LEAP2) Region Exhibits Inverse Agonist Activity toward the Ghrelin Receptor. *J Med Chem* **62**, 965-973 (2019).

4. Ferre G, *et al.* Structure and dynamics of G protein-coupled receptor-bound ghrelin reveal the critical role of the octanoyl chain. *Proc Natl Acad Sci U S A* **116**, 17525-17530 (2019).

5. Harris FM, Best KB, Bell JD. Use of laurdan fluorescence intensity and polarization to distinguish between changes in membrane fluidity and phospholipid order. *Biochim Biophys Acta* **1565**, 123-128 (2002).

6. Leyris JP, *et al.* Homogeneous time-resolved fluorescence-based assay to screen for ligands targeting the growth hormone secretagogue receptor type 1a. *Anal Biochem* **408**, 253-262 (2011).

7. Humphrey W, Dalke A, Schulten K. VMD: Visual molecular dynamics. *J Mol Graph Model* **14**, 33-38 (1996).
