# Supplementary material for: Allosteric modulation of ghrelin receptor signaling by lipids
Source: Nat Commun. 2021 Jun 24;12:3938. doi: 10.1038/s41467-021-23756-y (PMC8225672; doi:10.1038/s41467-021-23756-y)
Supplement: Supplementary file 3 — Reporting Summary [file 41467_2021_23756_MOESM3_ESM.pdf]

nature research

Corresponding author(s): Jean-Louis Banères

Last updated by author(s): Apr 26, 2021

# Reporting Summary

Nature Research wishes to improve the reproducibility of the work that we publish. This form provides structure for consistency and transparency in reporting. For further information on Nature Research policies, see our Editorial Policies and the Editorial Policy Checklist.

## Statistics

For all statistical analyses, confirm that the following items are present in the figure legend, table legend, main text, or Methods section.

| n/a | Confirmed | |
|---|---|---|
| ☐ | ☒ | The exact sample size (*n*) for each experimental group/condition, given as a discrete number and unit of measurement |
| ☐ | ☒ | A statement on whether measurements were taken from distinct samples or whether the same sample was measured repeatedly |
| ☐ | ☒ | The statistical test(s) used AND whether they are one- or two-sided<br>*Only common tests should be described solely by name; describe more complex techniques in the Methods section.* |
| ☒ | ☐ | A description of all covariates tested |
| ☐ | ☒ | A description of any assumptions or corrections, such as tests of normality and adjustment for multiple comparisons |
| ☐ | ☒ | A full description of the statistical parameters including central tendency (e.g. means) or other basic estimates (e.g. regression coefficient) AND variation (e.g. standard deviation) or associated estimates of uncertainty (e.g. confidence intervals) |
| ☐ | ☒ | For null hypothesis testing, the test statistic (e.g. $F$, $t$, $r$) with confidence intervals, effect sizes, degrees of freedom and $P$ value noted<br>*Give P values as exact values whenever suitable.* |
| ☒ | ☐ | For Bayesian analysis, information on the choice of priors and Markov chain Monte Carlo settings |
| ☒ | ☐ | For hierarchical and complex designs, identification of the appropriate level for tests and full reporting of outcomes |
| ☒ | ☐ | Estimates of effect sizes (e.g. Cohen's $d$, Pearson's $r$), indicating how they were calculated |

*Our web collection on statistics for biologists contains articles on many of the points above.*

## Software and code

Policy information about availability of computer code

| | |
|---|---|
| Data collection | Fluorescence and luminescence data were acquired and treated with the software of the dedicated instrument (Varian). Protein were purified using an Akta Pure system and data treated with the corresponding software (Unicorn 7.3). |
| Data analysis | Fluorescence and luminescence data were analyzed in GraphPad Prism 8, including statistical analyses. Competition curves were fitted using GraphPad Prism 8. Molecular dynamics data and structural views were generated using Gromacs 2020.3, VMD 1.9.3, Gnuplot 5.2, NAMD 2.13, MODELLER 9.19, CHARMM-GUI and MARTINI 2.2. |

For manuscripts utilizing custom algorithms or software that are central to the research but not yet described in published literature, software must be made available to editors and reviewers. We strongly encourage code deposition in a community repository (e.g. GitHub). See the Nature Research guidelines for submitting code & software for further information.

## Data

Policy information about availability of data

All manuscripts must include a data availability statement. This statement should provide the following information, where applicable:

- Accession codes, unique identifiers, or web links for publicly available datasets
- A list of figures that have associated raw data
- A description of any restrictions on data availability

The source data underlying Figs. 1a,b, 2d, 3b,c, 4a,b, 5a-d, 6a-f, 7c,d and Supplementary Figs. 1a-c, 2a-c, 3, 5c, 7, 8a,b, 9a-c, 10, 12a,b, 14, 15 and 17 are provided as a Source Data File. All other datasets supporting the findings of the study are available from the corresponding author on reasonable request. Evolved sortase is available from (Addgene #75144) and NW30 from DF/HCC plasmid depository (#HsCD00483426). The PDB files that were analyzed are published ones and can be found in the RCSB Protein Data Bank using the accession codes 6KO5 (https://www.rcsb.org/structure/6KO5) and 6VMS (https://www.rcsb.org/structure/6VMS).

# Field-specific reporting

Please select the one below that is the best fit for your research. If you are not sure, read the appropriate sections before making your selection.

[x] Life sciences [ ] Behavioural & social sciences [ ] Ecological, evolutionary & environmental sciences

For a reference copy of the document with all sections, see nature.com/documents/nr-reporting-summary-flat.pdf

# Life sciences study design

All studies must disclose on these points even when the disclosure is negative.

| | |
|---|---|
| Sample size | No sample size calculations were performed. Experiments were repeated 3 to 5 times to allow calculation of the mean and standard error of the mean. Sample size was chosen to obtain statistical significant values. |
| Data exclusions | No data was excluded from the analyses. |
| Replication | Independent experiments were repeated 3 times in the same laboratory to allow calculation of the mean and standard error of the mean. In the case of GTP turnover, the assays were repeated 5 times to obtain statistical significant values. The exact number of experiments is indicated in the legend of each figure. |
| Randomization | No randomization was attempted as it was not necessary for this kind experiments (no animal or clinical case/control study). |
| Blinding | No blinding was not performed as it was not necessary. In most cases, a given series of data were generated and analyzed by the same individual investigators. |

# Reporting for specific materials, systems and methods

We require information from authors about some types of materials, experimental systems and methods used in many studies. Here, indicate whether each material, system or method listed is relevant to your study. If you are not sure if a list item applies to your research, read the appropriate section before selecting a response.

## Materials & experimental systems

| n/a | Involved in the study | |
|---|---|---|
| [x] | [ ] | Antibodies |
| [x] | [ ] | Eukaryotic cell lines |
| [x] | [ ] | Palaeontology and archaeology |
| [x] | [ ] | Animals and other organisms |
| [x] | [ ] | Human research participants |
| [x] | [ ] | Clinical data |
| [x] | [ ] | Dual use research of concern |

## Methods

| n/a | Involved in the study | |
|---|---|---|
| [x] | [ ] | ChIP-seq |
| [x] | [ ] | Flow cytometry |
| [x] | [ ] | MRI-based neuroimaging |
